# MinION™ Nanopore Sequencing of Skin Microbiome 16S and 16S-23S rRNA Gene Amplicons

**DOI:** 10.3389/fcimb.2021.806476

**Published:** 2022-01-05

**Authors:** Miquel Rozas, François Brillet, Chris Callewaert, Bernhard Paetzold

**Affiliations:** ^1^R&D Department, S-Biomedic, Beerse, Belgium; ^2^Department of Experimental and Health Sciences, Universitat Pompeu Fabra (UPF), Barcelona, Spain; ^3^Center for Microbial Ecology and Technology, Ghent University, Ghent, Belgium

**Keywords:** skin microbiome, nanopore sequencing, MinION™, bacterial identification, skin mock community, 16S rRNA gene sequencing

## Abstract

Human skin microbiome dysbiosis can have clinical consequences. Characterizing taxonomic composition of bacterial communities associated with skin disorders is important for dermatological advancement in both diagnosis and novel treatments. This study aims to analyze and improve the accuracy of taxonomic classification of skin bacteria with MinION™ nanopore sequencing using a defined skin mock community and a skin microbiome sample. We compared the Oxford Nanopore Technologies recommended procedures and concluded that their protocols highly bias the relative abundance of certain skin microbiome genera, most notably a large overrepresentation of *Staphylococcus* and underrepresentation of *Cutibacterium* and *Corynebacterium*. We demonstrated that changes in the amplification protocols improved the accuracy of the taxonomic classification for these three main skin bacterial genera. This study shows that MinION™ nanopore could be an efficient technology for full-length 16S rRNA sequencing; however, the analytical advantage is strongly influenced by the methodologies. The suggested alternatives in the sample processing improved characterization of a complex skin microbiome community using MinION™ nanopore sequencing.

## Introduction

Precise characterization of the different human microbiomes is a critical first step toward understanding the host–microbe interactions in human health and disease (Lloyd-Price et al., 2016; Mohajeri et al., 2018; Rozas et al., 2021). Characterization of bacterial communities was revolutionized by the development of next-generation sequencing (NGS) techniques, which allowed microorganism discrimination to deeper taxonomic levels (Kchouk et al., 2017).

Due to its simplicity and reliability, the most standardized sequencing strategy to identify bacteria is based on the analysis of their 16S rRNA gene (Janda and Abbott, 2007). The 16S rRNA gene is essential in the bacterial domain and consists of ~1,500 bp containing 9 hypervariable regions (V1–V9) scattered among highly conserved sequences (Baker et al., 2003). All or some of 16S rRNA gene V1–V9 regions are amplified by polymerase chain reaction (PCR) using complementary primers to the conserved sequences (Boye et al., 1999). Resulting amplicons are sequenced and assigned to a bacterial taxonomic group by nucleotide sequence comparison with a reference nucleotide database (e.g., BLASTn) (Woo et al., 2008).

The first available sequencing technique, Sanger sequencing (Sanger et al., 1977; Chen et al., 2014), enabled 16S rRNA identification of bacterial clonal populations (Patel, 2001). Technical difficulties to maintain bacterial diversity when obtaining clonal populations, an effect known as the great plate count anomaly (Staley and Konopka, 1985; Lagier et al., 2015), limited the detectable species with Sanger sequencing (Fraher et al., 2012).

Overcoming these limitations, NGS techniques enabled the direct analysis of complex bacterial communities by parallel high-throughput generation of reads, providing faster and cheaper sequencing costs per sample (Metzker, 2010; Weinstock, 2012). However, the most popularized NGS technique Illumina is limited to short fragments (<600 bp) and does not allow sequencing of the entire 16S rRNA gene (Fan et al., 2006). Using NGS, taxonomic relative abundances are determined by analyzing subregions of the 16S rRNA gene, but the obtained results are biased by the selected subregion due to distinct primer binding affinities to each template (Polz and Cavanaugh, 1998; Kanagawa, 2003; Pollock et al., 2018). Therefore, it is not recommended to compare microbiome studies based on different 16S rRNA regions (Cai et al., 2013; Kennedy et al., 2014; Meisel et al., 2016; Graspeuntner et al., 2018).

In 2014, Oxford Nanopore Technologies (ONT) released a single-molecule sequencing technology that allows sequencing of DNA fragments without a theoretical length limit (Wang et al., 2015). High-throughput generation of reads is achieved in a pocket-sized portable device such as MinION™ (Lu et al., 2016). MinION™ instrument made nanopore sequencing widely accessible, allowing research centers to perform real-time data analysis, drastically reducing sequencing turnaround times, and lowering the cost per sequenced base (Branton et al., 2008). Nanopore technology allows entire 16S gene sequencing in samples with bacterial mixtures, overcoming at the same time, the main limitations of Sanger sequencing and NGS (Benítez-Páez et al., 2016). Nonetheless, nanopore sequencing still has higher base calling error rates than established NGS technologies (Jain et al., 2015).

In this study, a defined human skin bacterial genomic mock community and a skin microbiome sample were used to analyze the performance of ONT sequencing kits on taxonomic relative abundance and species-level determination. Recent studies focusing on other human microbiomes (e.g., gut) have already described bias of ONT sequencing kits toward certain genera and species (Heikema et al., 2020; Matsuo et al., 2021). To the best of our knowledge, no study has focused on analyzing the performance of ONT sequencing kits on the skin microbiome. Understanding the bias and limitations of ONT kits in taxonomizing bacteria of skin microbiome samples is crucial for future experimental designs and data interpretation (Meisel et al., 2016). Obtaining insights on the skin microbiome composition to genus and species level in skin health and disease will help to develop more effective prebiotic, probiotic, or drug therapies to treat skin diseases associated with microbiome dysbiosis.

## Materials and Methods

### Skin Microbiome Genomic Mix

Skin genomic mock community ATCC MSA-1005 was used in this study. It consists of an even mixture of six bacterial species each representing 16.7% [*Acinetobacter johnsonii* (ATCC 17909D-5), *Corynebacterium striatum* (ATCC 6940D-5), *Micrococcus luteus* (ATCC 4698D-5), *Cutibacterium acnes* (ATCC 11828D-5), *Staphylococcus epidermidis* (ATCC 12228D-5), and *Streptococcus mitis* (ATCC 49456D-5)].

### Skin Microbiome Standard

An artificial skin standard was created by mixing 72 extracted DNA samples of cheek skin swabs from Caucasian females and males of 12–25 years old. The study was approved by Innovapotek ethics committee for Health (Clinical investigation plan identification number P337119) and was performed following the Helsinki declaration of ethical principles for medical research involving human subjects (World Medical Association, 2013). Cheek swabs were collected, stored, and transported at -20°C using eNAT collection and transport system (Copangroup, USA). DNA was isolated and purified using DNAeasy 96 PowerSoil Pro Kit (Qiagen, UK) following its Quick-Start Protocol. In essence, samples were disrupted by mechanical bead-beating, and DNA was isolated and purified using silica membrane spin columns. A DNA skin standard was then obtained by combining 5 µl of each of the 72 extracted samples.

### 16S V1–V9 Nanopore Sequencing and Read Taxonomic Assignation

The 16S rRNA barcoded amplicons were produced in a single four-primer PCR reaction following Matsuo protocol (Matsuo et al., 2021). The following inner primers for amplification of V1–V9 of the 16S rRNA gene with complementary region underlined and anchor region were used: forward primer (27F) 5′-TTTCTGTTGGTGCTGATATTGCAGAGTTTGATCMTGGCTCAG-3′ and reverse primer (1492R) 5′-ACTTGCCTGTCGCTCTATCTTCCGGTTACCTTGTTACGACTT-3′. Barcoded outer primers containing the complementary anchor sequence to inner primers from PCR Barcoding Expansion Pack 1-96 EXP-PBC096 (Oxford Nanopore Technologies, UK) were used. DNA amplification was performed using Veriti 96 Well Fast Thermo Cycler in a reaction mix containing 200 nM of inner primers, 200 nM of outer primers, 12.5 µl of LongAmp polymerase mix, and 5 µl of template in a total volume of 25 µl. The cycling program used from Matsuo protocol was adapted to LongAmp polymerase. It consisted of 3-min denaturation at 95°C, 5 cycles (95°C–15 s, 55°C–15s, 65°C–90s), 30 cycles (95°C–15s, 62°C–15s, 65°C–90s), and a final extension step of 65°C for 2 min. Samples were also amplified using a KAPA HiFi HotStart PCR Kit KK2502 (Roche, Switzerland); using the same primer concentrations, mastermix was prepared following manufacturer recommendations and following Matsuo PCR conditions. PCR amplicons were run in 1% agarose gel in an electrophoresis chamber, pooled together and purified using DNA clean & concentrator kit (Zymoresearch, USA). Purified samples were then quantified with Accublue Broad Range dsDNA quantification kit (Biotium, USA) and further processed using SQK-LSK110 kit (Oxford Nanopore Technologies, UK). Library was sequenced using flow cell R9.4.1 (FLO-MIN106D) until the sample was exhausted or the desired number of reads was achieved. Basecalling was performed on MinION Mk1C using Guppy (version 5.0.13) with fast basecalling model and read filtering of min_score = 8. Epi2me (version v2021.09.09) was used to demultiplex the samples, filter reads retaining a size range of 1.2–1.8 kb, and assign the reads to its taxonomic group with default parameters using NCBI 16S database Coordinators (2016).

### 16S-23S Nanopore Sequencing and Read Taxonomic Assignation

NanoID kit is designed to produce 16S-23S amplicons of 2.5 kb. It contains forward primer complementary to 16S gene (27F): 5′-AGRRTTYGATYHTDGYTYAG-3′ and reverse primer complementary to 23S gene (23SR): 5′-AGTACYRHRARGGAANGR-3′. The 16S-23S amplicons were produced using NanoID kit (Shoreline Biome, USA) and following manufacturer’s instructions except for using DNA clean & concentrator (Zymoresearch, USA) instead of magnetic beads for the cleanup step. Library was prepared for sequencing using LSK-110 (Oxford Nanopore Technologies, UK) and sequenced using flow cell R9.4.1 (FLO-MIN106D) until the sample was exhausted or the desired number of reads was achieved. Basecalling was performed on MinION Mk1C using Guppy (version 5.0.13) with fast basecalling model and read filtering of min_score = 8. SBanalyser was used to demultiplex the samples, discard reads <200 bp, and assign the reads to its taxonomic group using Athena 16S-23S database (Coordinators, 2016).

### Illumina Sequencing and Operational Taxonomic Unit (OTU) Classification

The 16S rRNA hypervariable regions V1 and V3 were amplified and sequenced using Illumina MiSeq system by BaseClear B.V. (Netherlands). Initial quality assessment was based on data passing the Illumina Chastity filtering, and reads containing PhiX control signal were removed using a self-developed filtering protocol. Afterward, reads containing (partial) adapters were clipped (up to a minimum read length of 50 bp). A second quality assessment was performed based on the remaining reads using the FASTQC quality control tool (version 0.11.8). Paired-end sequence reads were collapsed into so-called pseudo reads using sequence overlap with USEARCH (version 9.2) (Edgar, 2010). Classification of these pseudo reads was performed based on the results of alignment with SNAP (version 1.0.23) (Zaharia et al., 2011) against the RDP database (version 11.5) (Cole et al., 2014) for bacterial organisms.

### Whole-Genome Shotgun Sequencing

Whole-genome shotgun sequencing was performed using Illumina HiSeq system by BaseClear B.V., Netherlands. Initial quality assessment was based on data passing the Illumina Chastity filtering, and reads containing PhiX control signal were removed using a self-developed filtering protocol. Afterward, reads containing (partial) adapters were clipped (up to a minimum read length of 50 bp). A second quality assessment was performed based on the remaining reads using the FASTQC quality control tool (version 0.11.8). Alignment-based filtering was performed by aligning the Illumina reads against the reference sequence using BBmap (version 38.79). Kraken2 (Wood et al., 2019) (version 2.0.8) was used to taxonomically classify the metagenomic reads based on a reference database enriched with skin-specific genomes. Species- and genus-level relative abundance profiles were obtained using Bracken (version 2.6.0) (Lu et al., 2017).

### Statistical Analysis

The Pearson correlation coefficient (r) was computed to compare the bacterial compositions analyzed by the different sequencing methods. Statistical significance was defined by a two-tailed P-value <0.05 [performed with Prism 9.2.0 (GraphPad Software, Inc., La Jolla, CA, USA)]. Similarity matrices (Pearson correlation) and hierarchical clustering (one minus Pearson correlation) to compare the different sequencing methods were performed with MORPHEUS (https://software.broadinstitute.org/morpheus) and edited with Prism 9.2.0. Simple linear regression (R squared) was computed to compare log 10 reads and percentage of GC (%GC) in the samples among the different sequencing methods (Prism 9.2.0).

## Results

### Genomic Skin Mock Community Taxonomic Classification

**Figure 1:**
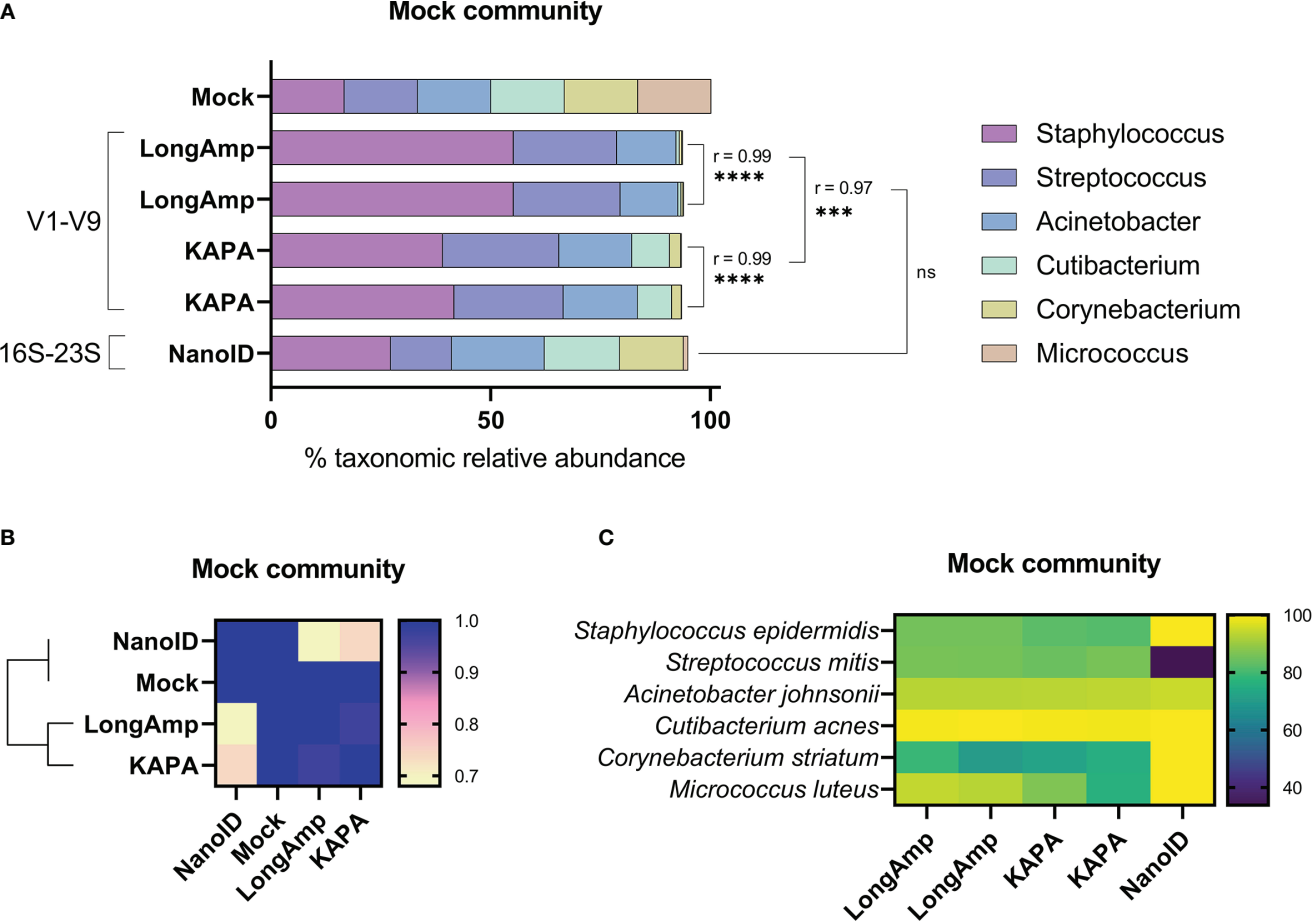
Testing of different amplification methodologies for MinION™ sequencing of human mock skin microbial communities. **(A)** Comparison of taxonomic profiles of classified reads of the mock community. The Pearson coefficient (r) between sequencing methods was computed to highlight a significant correlation between samples and/or methodologies. ns, not significant; ***P ≤ 0.001; ****P ≤ 0.0001. **(B)** Similarity matrix and hierarchical clustering of the methodologies based on their relative abundance profiles. **(C)** Heat map showing the percentage of classified reads to the correct species between the sequencing methods in the mock community.

The aim of our study was to verify whether nanopore sequencing is an accurate technique to investigate the skin microbiome. In order to do so, we aimed to generate and sequence a library of V1–V9 16S rRNA gene amplicons using ONT library prep kits and the defined genomic skin mock community. Duplicates of V1–V9 amplicons were successfully generated for the mock community using a four-primer PCR. The library was sequenced and basecalled with MinION Mk1C, generating more than 25,000 reads in the length range of 1.2–1.8 kb with a quality score ≥8 that afterward were classified to its taxonomic group with Epi2me (**Supplementary Table S1**). Duplicates statistical comparison and taxonomic relative abundances obtained using LongAmp polymerase are shown in **Figure 1A**. Statistically significant similarities (Pearson correlation) were found in the genus relative abundances across the duplicates. Each genus in the mock community was expected to be 16.7%. We found in our analysis that *Staphylococcus* (~55.2%) and *Streptococcus* (~23.9%) were respectively highly and mildly overrepresented. *Acinetobacter* (~13.3%) was slightly underrepresented and *Cutibacterium* (~0.7%), *Corynebacterium* (~0.5%), and *Micrococcus* (~0.1%) were highly underrepresented. Moreover, ~6.5% of the classified reads were not assigned to any of these six genera.

After assessing the possibilities causing this large bias on some genera, we found that the three underrepresented genera have higher GC content (*Cutibacterium* 60.1%, *Corynebacterium* 59.3%, and *Micrococcus* 73.1%) compared to the overrepresented genera (*Staphylococcus* 32.2% and *Streptococcus* 40.1%). We hypothesized that the polymerase recommended in ONT protocol LongAmp could be one of the reasons of the underrepresentation of genera with high GC content. Therefore, we assessed the performance of KAPA, a polymerase widely used for NGS applications, to estimate bacterial relative abundance for the mock community. Applying the same pipeline but using KAPA instead of LongAmp polymerase, more than 25,000 reads in the length range of 1.2–1.8 kb with a quality score ≥8 were obtained, and Epi2me assigned them to taxonomic groups (**Supplementary Table S1**). Duplicates statistical comparison and taxonomic relative abundances obtained using KAPA are shown in **Figure 1A**. Statistically significant similarities (Pearson correlation) have been found in the genus relative abundances across the duplicates. Comparing to the previous results obtained using LongAmp, relative abundances obtained for *Staphylococcus (*~40.4%), *Cutibacterium* (~8.2%), *Corynebacterium (*~2.3%), and *Acinetobacter* (~16.7%) were significantly correlated but closer to the expected in the mock community (16.7%). *Streptococcus* (~25.7%) and *Micrococcus* (~0.2%) relative abundances were not affected by the change of polymerase neither the percentage of unclassified reads to any of these genera (~6.5%). Overall, closer relative abundances to the mock community were obtained using KAPA, but the obtained relative abundances were still poorly representing the mock community.

Differential primer affinities to 16S rRNA genes have been described to produce bias when determining relative abundances in mixed bacterial samples (Sipos et al., 2007). When comparing the previously used 1492R primer to the 16S gene sequences present in the mock community using NCBI database, we observed that 1492R does not completely bind any of the genera. To see if primers with affinity to a broader range of bacteria would improve the relative taxonomic abundances obtained, we used NanoID kit from Shoreline Biome. NanoID uses a degenerated version of 27F primer and a reverse degenerated primer complementary to 23s gene, which is ~1 kb downstream than the binding site of 1492R. The 16S-23S amplicons were successfully generated following NanoID guidelines and sequenced and basecalled with MinION Mk1C. Sbanalyzer filtered reads below 200 bp and successfully assigned more than 95.000 reads to a bacterial taxonomic level (**Supplementary Table S1**). The obtained taxonomic relative abundances and statistical comparisons are shown in **Figure 1A**. Comparing to V1–V9 results obtained using KAPA and LongAmp polymerase, NanoID shows non-significant similarities. Relative abundances obtained with NanoID for *Staphylococcus* (~27.3%), *Cutibacterium* (~17.2%), *Corynebacterium* (~14.5%), and *Streptococcus* (~13.9%) were considerably closer to the mock community. *Acinetobacter* (~21.1%) estimation was less accurate, and *Micrococcus* (~1.1%) was improved but still largely underrepresented. Slightly lower percentage of reads (~5%) were not classified to any of the genera from the mock community. Even though *Micrococcus* was largely underrepresented, NanoID showed a better overall performance to determine bacterial relative abundance in the mock community than the previously tested protocols (see similarity matrix, **Figure 1B**).

Another relevant parameter to analyze is the percentage of reads in each genus that were classified to the proper species. All V1–V9 reads were classified to a species, while a small fraction of 16S-23S reads were classified to a species level. In the mock community, each genus is exclusively composed of a single bacterial species and the number of reads assigned to the correct species were analyzed to calculate the percentage of correctly identified species in each genus (**Figure 1C**). No differences were observed in V1–V9 sequencing runs between LongAmp and KAPA and are shown together as V1–V9. The obtained percentages of correctly identified species for V1–V9 and 16S-23S were respectively the following: *Staphylococcus epidermidis* (~83.6%, ~99.9%), *Cutibacterium acnes* (~99.5%, ~100%), *Corynebacterium striatum (*~74%, ~99.9%), *Streptococcus mitis* (~85.4%, ~33.8%), *Acinetobacter johnsonii* (~93.3%, ~94.6%). This value was not determined for *Micrococcus luteus* due to the low number of reads obtained. Overall, 16S-23S amplicons resulted in a more accurate species determination with the sole exception of *Streptococcus mitis*, for which ~66.1% of the reads were classified as *Streptococcus pneumoniae*.

### Skin Standard Taxonomic Classification

**Figure 2:**
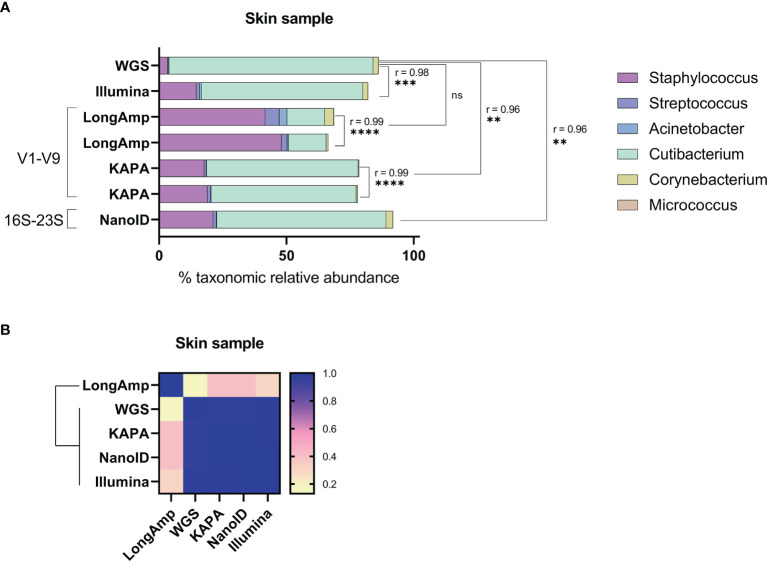
Testing of different amplification methodologies for MinION™ sequencing of human skin sample microbial communities. **(A)** Comparison of taxonomic profiles of classified reads of the skin sample communities. The Pearson coefficient (r) between sequencing methods was computed to highlight a significant correlation between samples and/or methodologies. ns, not significant; **P ≤ 0.01; ***P ≤ 0.001; ****P ≤ 0.0001. **(B)** Similarity matrix and hierarchical clustering of the methodologies based on their relative abundance profiles.

We tested if the described observations in the skin mock community would also apply to a real skin microbiome sample. First, since our skin microbiome standard had an unknown composition, we analyzed the bacterial relative abundance by sequencing its V1–V3 16S rRNA region with Illumina MiSeq and by shotgun whole-genome sequencing (WGS) sequencing (**Figure 2A**). The relative abundances obtained with MiSeq and WGS were, respectively, the following: *Staphylococcus* (~14.6%, ~3.4%), *Cutibacterium* (~63.3%, ~80.1%), *Corynebacterium* (~2%, ~2%), *Streptococcus* (~1.3%, ~0.4%), *Acinetobacter* (~0.8%, ~0.1%), and *Micrococcus* (~0.1%, ~0.1%). Then, we processed the skin microbiome standard with the three conditions previously tested (V1–V9 with LongAmp, V1–V9 with KAPA, and 16S-23S with NanoID). We generated, 16,617 and 2,610 reads for LongAmp duplicates, more than 15,000 for KAPA duplicates and more than 78,000 reads for NanoID (**Supplementary Table S1**). Afterward, with its corresponding software and database, reads were assigned to a taxonomic group. The obtained taxonomic relative abundances and statistical comparisons between duplicates and different methods are shown in **Figure 2A**. Statistically significant similarities (Pearson correlation) have been found in the genus relative abundances across the duplicates. Relative abundances obtained using LongAmp, KAPA, and NanoID were respectively the following: *Staphylococcus* (~44.9%, ~18.4%, ~21.2%), *Cutibacterium* (~14.8%, ~58.2%, ~66.5%), *Corynebacterium* (~2.1%, ~0.4%, ~2.7%), *Streptococcus* (~3.9%, ~1.1%, ~1.3%), *Acinetobacter* (~1.8%, ~0.16%, ~0.15%), and *Micrococcus* (<0.1%, <0.1%, <0.1%). As can be seen in **Figure 2A** and taking WGS as reference, *Staphylococcus* was largely overrepresented when using the recommended polymerase by ONT kits LongAmp and to a lesser extent still overrepresent for all other techniques. Except for LongAmp polymerase, the other techniques have significant Pearson correlation compared to WGS. The similarity matrix (**Figure 2B**) shows similarities for all the techniques except for LongAmp. Altogether, these results suggest that the biases observed in the mock community also apply to a real skin microbiome sample, and this bias can be reduced by changing the polymerase or primers used.

## Discussion

**Figure 3:**
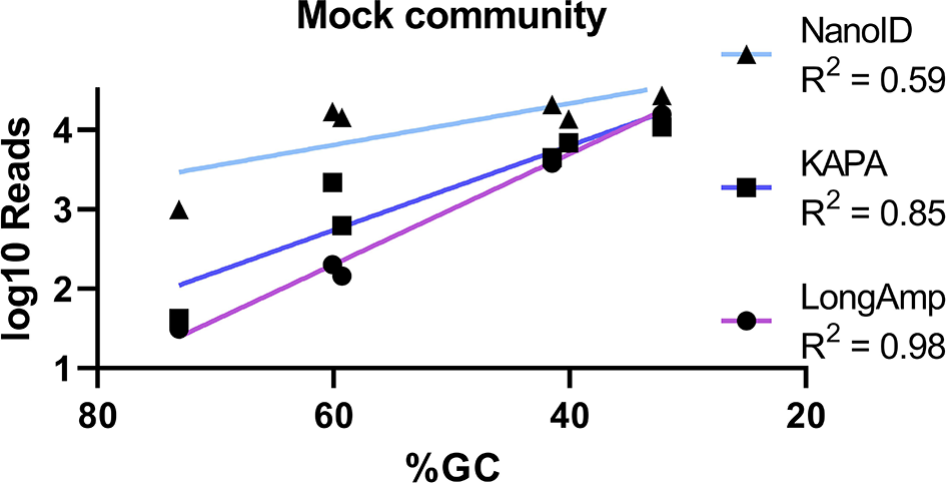
Basic linear regression analysis used to correlate the GC content (%) of mock community skin genera in sequenced samples (x-axis) compared to the number of reads in the MinION™ sequenced samples (y-axis).

Nanopore is revolutionizing sequencing in laboratories by generating high-throughput reads that can be analyzed in real time, reducing total processing time and sequencing costs per sample. Nevertheless, its lower basecall accuracy (85%–93%) and described biases toward certain genera and species in complex bacterial samples (Heikema et al., 2020; Matsuo et al., 2021) urged us to investigate if ONT is ready to be used in skin microbiome analysis. Using a defined genomic skin mock community, we show that recommended polymerase (LongAmp) and 16S primer sequences in ONT kits have a strong bias toward the most prevalent skin bacterial genera and toward low GC-content bacteria (**Figure 3**). Furthermore, we show that using a different polymerase (KAPA) and primer selection (NanoID) can reduce this bias and improve the overall results. These improvements were demonstrated on a bacterial skin mock community and confirmed in a real skin microbiome sample.

Upon studying the performance of the recommended polymerase by Nanopore, LongAmp, on the skin mock community, we observed a strong bias toward certain genera (**Figure 1A**). *Staphylococcus* was highly and *Streptococcus* mildly overrepresented, while *Cutibacterium*, *Corynebacterium*, and *Micrococcus* were strongly underrepresented. Overrepresented and underrepresented genera had respectively low and high genomic GC contents. It has been described that polymerase performance can be negatively influenced by a high GC content (Dabney and Meyer, 2012). To assess this problem, we tested KAPA, a widely used polymerase for NGS studies that has improved performance on GC-rich templates (Oyola et al., 2012). Using KAPA, *Staphylococcus* abundance overrepresentation decreased and *Cutibacterium*, *Corynebacterium*, and *Acinetobacter* abundances increased, obtaining for the four genera closer bacterial relative abundances to the defined mock community. *Streptococcus* overrepresentation and *Micrococcus* vast underrepresentation did not improve with KAPA polymerase (**Figure 1A**). *Micrococcus* is the genus with the highest GC content (73.1%) in the mixture that makes it a complicated target. It is known that GC-rich DNA double strands require higher energy for strand dissociation, reducing their availability for primer binding and resulting in lower PCR amplification (Laursen et al., 2017). Other factors such as the differences in 16S gene copy number mean in the genomes of *Staphylococcus epidermidis* (5.9), *Streptococcus mitis* (3.9), *Acinetobacter johnsonii* (7), *Cutibacterium acnes* (3.1), *Corynebacterium striatum* (4), and *Micrococcus luteus* (2.1) can influence the 16S amplicon amounts produced on PCR (Stoddard et al., 2015).

Another important variable described to cause bias in amplification of mixed genomic samples is the primer binding affinity to each target, which decreases with lower sequence similarity (Sipos et al., 2007). The NCBI database shows differences in sequence similarity of primer 1492R among the genera in the used mock community. A different primer pair was used in an attempt to improve the obtained relative abundance. These primers, included in the NanoID kit, were designed by Shoreline biome to have higher affinity to a broader variety of bacterial species. It is important to notice that the polymerase used by NanoID kit is not disclosed by the provider. When using NanoID kit to amplify 16S-23S region, *Staphylococcus*, *Cutibacterium*, *Corynebacterium*, and *Streptococcus* relative abundances were closer to the mock community than the ones obtained with V1–V9 amplifications. NanoID has a slightly poorer performance on determining *Acinetobacter* relative abundance than V1–V9 region, and even if NanoID performed better for *Micrococcus*, this genus was still vastly underrepresented (**Figure 1A**). Primers used in NanoID are a degenerated version of 27F primer, and instead of the 1492R primer, it contains a degenerated primer complementary to the downstream 23S rRNA gene. Degenerated primers have already been shown to be a good alternative when targeting a broad taxonomic range of bacteria (Boye et al., 1999). Overall, NanoID obtained better relative abundances than any of the tested polymerases amplifying the region V1–V9, even though it is not clear if the improvement on relative abundances using NanoID is caused by their degenerated primers, their undisclosed polymerase, or a combined effect of both.

V1–V9 sequencing data were analyzed with Epi2me assigning all reads to species level, a much larger percentage compared to NanoID kit data analyzed with Sbanalyzer. This large difference on percentages of assigned reads is due to software restrictions for species-level read classification. Default criteria for read assignation to species level is less restrictive on Epi2me than on Sbanalyzer, resulting in a higher percentage of false-positive results for species identification and making Sbanalyzer species results more reliable. This observation is consistent for all the genera except for *Streptococcus* where species *S. pneumoniae*, a well-recognized human pathogen, accounted for more than half of the genus assigned reads. Besides, it is important to keep in mind that NanoID generates 16S-23S amplicons, which are longer than the V1–V9 amplicons, allowing a more precise read discrimination to species level (Mendoza et al., 1998). Differentiation at species level of a skin commensal such as *Staphylococcus epidermidis* from the skin pathogen *Staphylococcus aureus* can be crucial in diagnostic procedures (Hauser et al., 1985; Jukes et al., 2010). Therefore, depending on the aim of the study and the impact of false positives, more permissive or restrictive analysis criteria should be chosen accordingly.

In order to assess if the described relative abundance biases for the mock community using ONT kits also apply to real skin samples, a real skin microbiome sample was analyzed. Since the actual taxonomic composition of the skin sample was unknown, shotgun whole-genome sequencing (WGS) and V1–V3 MiSeq were performed as a means of comparison. WGS has been described as the most accurate technique for determining bacterial taxonomic relative abundances on skin samples, while V1–V3 MiSeq gives a close estimation (Meisel et al., 2016). Taking WGS as the more realistic estimation, LongAmp performed very poorly on determining *Cutibacterium* and *Staphylococcus* abundances compared to all other methods. V1–V9 amplified with KAPA, NanoID, and V1–V3 MiSeq show similar levels of *Cutibacterium* underrepresentation and *Staphylococcus* overrepresentation. V1–V3 MiSeq and NanoID gave a better approximation of *Corynebacterium* than V1–V9 methods. For the rest of the genera in the mock community, it is difficult to assess the performance of the different conditions, since each of these genera represents less than 0.5% in the WGS data. Among the different strategies followed to analyze the skin sample with nanopore sequencing, NanoID produced the closest results to WGS, which also were very similar to the ones obtained with V1–V3 MiSeq. Nonetheless, it should be noticed that WGS data were obtained with a *k*-mer approach that can have sequencing errors that lead to wrong taxonomic assignations. This can result in bacterial diversity overestimation and bacterial relative abundance underestimation. A metagenome-assembled genome (MAG) approach for WGS could provide a more realistic data to be used as a benchmark to evaluate the bias in the different techniques more precisely (Pasolli et al., 2019). Therefore, some of the biases described in this study could be larger than observed. Overall, we have shown that the observed biases in a defined skin mock community also apply to a real skin microbiome sample and can also be reduced by using alternative polymerases and primers.

Nanopore sequencing is a relatively new sequencing technology with many advantages but also with some limitations such as a higher error rate than other sequencing platforms. ONT improvements in sequencing flow cells with R10 chemistry and improvements in newer versions of Guppy basecaller can produce more accurate data. These improvements can have a big influence in species and strain determination when base variants are used to assign taxonomy. Nonetheless, we observed a strong bias at higher taxonomic levels on the main skin genera abundances when recommended polymerase (LongAmp) and 16S primers by ONT kits were used. We suggested an alternative polymerase (KAPA) and primers (NanoID) that generated better results in a defined skin mock community and a skin microbiome sample. However, variables such as polymerase and primers used, PCR conditions, and bioinformatic analysis should be further improved to obtain more reliable data with this technology. Therefore, we encourage the scientific community for further improving the protocols for skin microbiome sequencing using MinION. Once this issue is addressed, nanopore sequencing will allow precise, faster, and cheaper generation of data in skin microbiome studies.

## Data Availability Statement

The datasets presented in this study can be found in online repositories. The names of the repository/repositories and accession number(s) can be found below: BioProject ID PRJNA783735.

## Ethics Statement

The studies involving human participants were reviewed and approved by Innovapotek ethics committee for Health–Clinical investigation plan identification number P337119. Written informed consent to participate in this study was provided by the participants’ legal guardian/next of kin.

## Author Contributions

MR and BP designed the study. MR performed the experiments. MR, BP, and FB analyzed the data. MR, BP, FB, and CC edited and reviewed the article. All authors contributed to the article and approved the submitted version.

## Funding

All research was funded by S-Biomedic.

## Conflict of Interest

The authors declare that the research was conducted in the absence of any commercial or financial relationships that could be construed as a potential conflict of interest.

## Publisher’s Note

All claims expressed in this article are solely those of the authors and do not necessarily represent those of their affiliated organizations, or those of the publisher, the editors and the reviewers. Any product that may be evaluated in this article, or claim that may be made by its manufacturer, is not guaranteed or endorsed by the publisher.
