# Supplementary material for: MinION™ Nanopore Sequencing of Skin Microbiome 16S and 16S-23S rRNA Gene Amplicons
Source: Front Cell Infect Microbiol. 2022 Jan 5;11:806476. doi: 10.3389/fcimb.2021.806476 (PMC8766866; doi:10.3389/fcimb.2021.806476)
Supplement: Supplementary file 1 [file Table_1.docx]

| Mock community |  |  |  |  |  |  |  |
| --- | --- | --- | --- | --- | --- | --- | --- |
| Method | **No. Classified reads** | ***Staphylococcus*** | ***Cutibacterium*** | ***Corynebacterium*** | ***Acinetobacter*** | ***Streptococcus*** | ***Micrococcus*** |
| V1-V9 LongAmp.1 | 29,623 | 16,347 | 202 | 140 | 3,897 | 7,204 | 34 |
| V1-V9 LongAmp.2 | 26,946 | 14,865 | 200 | 147 | 3,640 | 6,340 | 28 |
| V1-V9 KAPA.1 | 27,698 | 11,553 | 2,164 | 589 | 4,681 | 6,879 | 38 |
| V1-V9 KAPA.2 | 25,456 | 9,948 | 2,186 | 650 | 4,218 | 6,745 | 46 |
| NanoID | 97,950 | 26,713 | 16,810 | 14,226 | 20,566 | 13,584 | 986 |
| Skin Standard |  |  |  |  |  |  |  |
| Method | **No. Classified reads** | ***Staphylococcus*** | ***Cutibacterium*** | ***Corynebacterium*** | ***Acinetobacter*** | ***Streptococcus*** | ***Micrococcus*** |
| WGS | 17,078,491 | 579,005 | 13,686,529 | 346,759 | 20,150 | 73,456 | 21,091 |
| V1-V3 Illumina | 14,145 | 2,153 | 8,957 | 1,230 | 11 | 183 | 8 |
| V1-V9 LongAmp.1 | 16,617 | 7,992 | 2,452 | 127 | 90 | 370 | 0 |
| V1-V9 LongAmp.2 | 2,610 | 1,087 | 388 | 91 | 78 | 146 | 1 |
| V1-V9 KAPA.1 | 15,986 | 3,036 | 9,099 | 94 | 32 | 205 | 1 |
| V1-V9 KAPA.2 | 18,290 | 3,248 | 10,886 | 53 | 21 | 154 | 2 |
| NanoID | 78,378 | 16,607 | 52,114 | 2,122 | 120 | 1,058 | 15 |

Table 1. Reads assigned to each genera for the different methods tested in the mock community and skin standard.
